# *Sulfobacillus thermosulfidooxidans* strain Cutipay enhances chalcopyrite bioleaching under moderate thermophilic conditions in the presence of chloride ion

**DOI:** 10.1186/s13568-014-0084-1

**Published:** 2014-12-10

**Authors:** Roberto A Bobadilla-Fazzini, Maria Paz Cortés, Alejandro Maass, Pilar Parada

**Affiliations:** BioSigma S.A., Parque Industrial Los Libertadores, Lote 106, Colina, Santiago de Chile, Chile; Laboratory of Bioinformatics and Mathematics of the Genome, Center for Mathematical Modeling (UMI2807-CNRS) and FONDAP Center for Genome Regulation, Santiago de Chile, Chile; Faculty of Mathematical and Physical Sciences, University of Chile, Santiago de Chile, Chile; Department of Mathematical Engineering, Center for Mathematical Modeling (UMI2807-CNRS) and FONDAP Center for Genome Regulation, Santiago de Chile, Chile

**Keywords:** Copper, Chalcopyrite, Bioleaching, Chloride ion, Chloride resistance, Sulfobacillus

## Abstract

Currently more than 90% of the world’s copper is obtained through sulfide mineral processing. Among the copper sulfides, chalcopyrite is the most abundant and therefore economically relevant. However, primary copper sulfide bioleaching is restricted due to high ionic strength raffinate solutions and particularly chloride coming from the dissolution of ores. In this work we describe the chalcopyrite bioleaching capacity of *Sulfobacillus thermosulfidooxidans* strain Cutipay (DSM 27601) previously described at the genomic level (Travisany et al. (2012) Draft genome sequence of the Sulfobacillus thermosulfidooxidans Cutipay strain, an indigenous bacterium isolated from a naturally extreme mining environment in Northern Chile. J Bacteriol 194:6327–6328). Bioleaching assays with the mixotrophic strain Cutipay showed a strong increase in copper recovery from chalcopyrite concentrate at 50°C in the presence of chloride ion, a relevant inhibitory element present in copper bioleaching processes. Compared to the abiotic control and a test with *Sulfobacillus acidophilus* DSM 10332, strain Cutipay showed an increase of 42 and 69% in copper recovery, respectively, demonstrating its high potential for chalcopyrite bioleaching. Moreover, a genomic comparison highlights the presence of the 2-Haloacid dehalogenase predicted-protein related to a potential new mechanism of chloride resistance in acidophiles. This novel and industrially applicable strain is under patent application CL 2013–03335.

**Electronic supplementary material:**

The online version of this article (doi:10.1186/s13568-014-0084-1) contains supplementary material, which is available to authorized users.

## Introduction

According to the Chilean Copper Commission (COCHILCO, [2014]), Chile is the largest copper producer where hydrometallurgy represented 33% of the 2013 local copper production. Copper sulfides processing accounts for more than 90% of world’s production. Among the copper sulfides, chalcopyrite constitutes the vast majority of copper resources and reserves, and therefore represents a mineralogical species of great economical importance (Doebrich [2009]).

Within the available hydrometallurgical technologies heap leaching is one of the most used, particularly for copper oxides and secondary copper sulfides. The detection and identification of microorganisms able to live and reproduce under the acidic conditions of the heap leaching process, and moreover the biotechnological innovations related to their potential use as catalysts for primary copper sulfide bioleaching, are topics of major interest. Different studies have shown the enhancing effect of mesophilic (Bobadilla Fazzini et al. [2011]), moderate thermophilic (Stott et al. [2003]) and thermophilic (Konishi et al. [1999]) microorganisms for chalcopyrite dissolution compared to abiotic assays. However, most of these studies were performed under lab-scale ideal conditions, not taking into account industrial-scale process variables such as high ionic strength solutions.

The application of heap bioleaching for copper recovery is severely limited due to fresh-water scarcity as in northern Chile, or the saline drains in Western Australia, due to solutions of high ionic strength and concentrations of elements coming from the ore minerals dissolution such as atacamite (Cu_2_Cl(OH)_3_) that releases chloride ions, which are inhibitory for many acidophilic iron-oxidizing microbes. In our estimation, during 2013 about 700 million tons of ore with an average copper grade of 0.2% could not be bioleached worldwide due to the inhibitory effect of high ionic strength raffinate solutions over acidophiles. The most studied acidophilic species is *Acidithiobacillus ferrooxidans*, and its sensitivity to low chloride concentration has been described long before, attributed to the loss of the cell outer layer integrity (Lawson et al. [1995]). In the case of the widely distributed acidophilic species *Leptospirillum ferriphilum*, ferrous ion biooxidation is completely inhibited by 12 g/L chloride (Gahan et al. [2010]). Moreover, prolonged exposure to sodium chloride demonstrated no significant adaptation of mixed chemolithotrophic iron-oxidizing cultures, indicating the lack of an acquired resistance through adaptation (Shiers et al. [2005]). Nevertheless, resistant species exist such as *Thiobacillus prosperus*, a halotolerant acidophile that requires chloride for growth (Nicolle et al. [2009]) or the marine *Sulfobacillus* sp. TPY able to tolerate 2% (w/v) NaCl (Wang et al. [2012]).

With respect to the biochemical mechanism of bacterial chloride resistance, few studies are available particularly on acidophiles. A recent study showed that NaCl stress in the moderate thermophiles *Acidimicrobium ferrooxidans* and *Acidithiobacillus caldus* is tackled by cell membrane adaptation and potential osmoprotectant aminoacid accumulation (Zammit et al. [2012]), giving an insight on specific chloride resistance genes.

Based on these facts, a search for chloride resistant iron-oxidizing acidophilic strains is a relevant research topic. In this study we describe the chalcopyrite bioleaching capacity of *Sulfobacillus thermosulfidooxidans* strain Cutipay (DSM 27601) in the presence of chloride ion. Strain Cutipay was previously described with novel copper and arsenic resistance capacities at the genomic level (Travisany et al. [2012]).

## Material and methods

### Strains and culture conditions

*Sulfobacillus thermosulfidooxidans* DSM 27601 strain Cutipay was isolated from an ore deposit in northern Chile. *Sulfobacillus acidophilus* DSM 10332 was obtained from the DSMZ collection. Both strains were cultivated in shake flasks at 50°C in 9 K medium at pH 1.6 (Bobadilla Fazzini et al. [2011]), including the addition of 0.25 g/L yeast extract for mixotrophic growth on ferrous. Microorganisms were inoculated at 1% v/v. The cell number was determined by chamber counting under phase-contrast microscope (Thoma Chamber, depth 0.010 mm) with at least four replicate counts.

### Minimum inhibitory concentration assays

Minimum inhibitory concentration assays (MIC) were performed in 6 well plates with 5 mL of 9 K medium at pH 1.6 for mixototrophic growth on ferrous, inoculated with 1,00E + 07 cells/mL for each strain separately. Assays were incubated with agitation at 50°C for 5 d with a top film to prevent evaporation. Different concentrations of chloride (tested as NaCl or KCl), Cu(II) (added as CuSO_4_) and As(III) (added as As_2_O_3_) were tested. The lowest concentration at which no growth or iron oxidation was observed corresponds to the MIC that were assayed at least in triplicate.

### *In silico* determination of orthologous protein clusters

The genome sequences and annotation of the predicted proteomes for *Sulfobacillus acidophilus* DSM10332 (GenBank IDs:NC_016884.1 and NC_016888.1) and *Sulfobacillus* sp. TPY (GenBank ID:NC_015757.1) were obtained from the GenBank database. For *Sulfobacillus thermosulfidooxidans* strain Cutipay, predicted proteome sequence was obtained from an in-house annotation of its genome (GenBank ID: ALWJ01000000) as described previously (Travisany et al. [2012]). Using these three predicted proteomes, orthologous protein clusters were determined using ORTHOMCL v1.4 (Li et al. [2003]) with default parameters.

### Bioleaching tests

Bioleaching tests were done in shake flasks, as a minimum in duplicate. Each flask contained 100 mL 9 K medium (pH 1.6), as described above, and supplemented with 1.5 g/L Fe(III) and 2.5 g/L Fe(II) with 1% w/v 300 Tyler mesh sieved chalcopyrite concentrate (85.5% chalcopyrite representing more than 99% of total Cu in the assay (Additional file 1: Table S1)) and 3 g/L chloride (as sodium chloride). Each flask was inoculated with *Sulfobacillus thermosulfidooxidans* DSM 27601 or *Sulfobacillus acidophilus* DSM 10332 (1 × 10^7^ cell/mL each). A non-inoculated control was included. Shake flasks were incubated for 27 d at 50°C with 120 rpm agitation. Each week cell number, Fe(II) by *o*-phenantroline method, total iron and Cu(II) by atomic absorption spectrometry were assayed. Sampling was performed allowing the suspended concentrate to settle, and filtering the supernatant to avoid any solids (Grade 5C filters, Advantec, Japan). Evaporation was compensated with pure water before each sampling time.

## Results

In order to determine the phenotypic properties of *Sulfobacillus thermosulfidooxidans* strain Cutipay (DSM 27601) related to heavy metals and chloride resistance, MIC assays were performed and compared to a collection *Sulfobacillus* strain. The results are summarized in Table 1.

**Table 1:** **Minimum Inhibitory Concentrations (MIC) for inhibitory elements chloride, copper and arsenic**

Moderate thermophilic species	Chloride (KCl)	Chloride (NaCl)	Cu(II)	As(III)
	[ppm]			
*Sulfobacillus thermosulfidooxidans* Cutipay (DSM 27601)	10,000	5,000	3,000	<100
*Sulfobacillus acidophilus* (DSM 10332)	3,000	1,000	1,000	1,000

**Figure 1:**
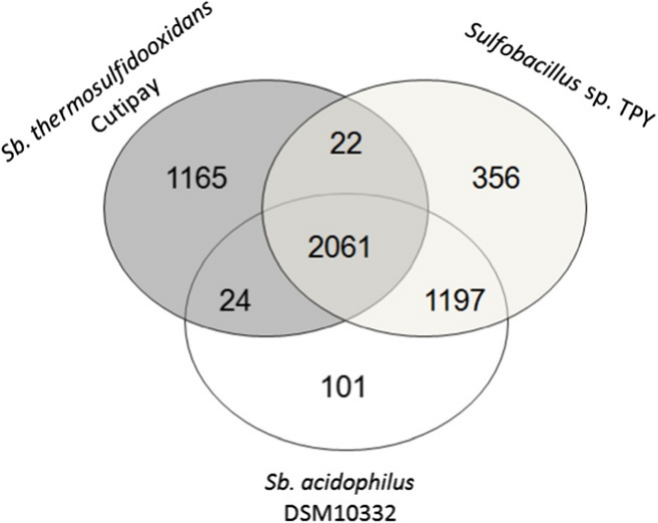
**Venn diagram of orthologous genome coded protein clusters for chloride-resistant strains** ***Sulfobacillus*** **sp. TPY and** ***Sulfobacillus thermosulfidooxidans*** **Cutipay, and chloride-sensitive strain** ***Sulfobacillus acidophilus*** **DSM 10332.** The diagram shows the number of shared and unique clusters between the three strain genomes.

Significant differences in chloride MIC using sodium or potassium salts were observed, requiring two to three times of potassium chloride to reach total inhibition compared to the sodium salt. Cutipay showed a higher resistance to chloride (three to five times depending the counter-ion used) and copper (three times) and a lower resistance to arsenic (ten times) compared to *Sulfobacillus acidophilus* DSM 10332. To understand these phenotypes, a whole genome comparison of selected *Sulfobacillus* species was performed looking for specific chloride resistance genes. The genomes of *Sulfobacillus thermosulfidooxidans* strain Cutipay and *Sulfobacillus* sp. TPY, as representatives of chloride-resistant species, were compared together and against *Sulfobacillus acidophilus* DSM10332, a chloride sensitive strain (Figure 1).

The three strains have 2061 of coded proteins in common. An exclusive cluster of 22 proteins was shared between both chloride resistant strains. An interesting protein annotated as Haloacid dehalogenase (sequence identifier gi|339628570|ref|YP_004720213.1|) was recognized. This protein belongs to the haloacid dehalogenase-like (HAD) superfamily that includes the 2-Haloacid dehalogenase that catalyzes the dechlorination of small organic chloro-acids.

**Figure 2:**
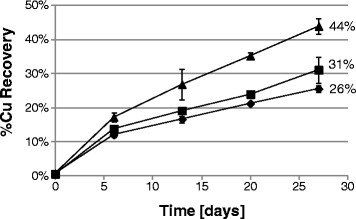
**Copper recovery in chalcopyrite bioleaching tests in the presence of chloride ion (3 g/L), incubation at 50°C and addition of** ***Sulfobacillus thermosulfidooxidans*** **DSM 27601 strain Cutipay (▲);** ***Sulfobacillus acidophilus*** **DSM 10332 (♦).** A control without inoculation was included (■).

**Figure 3:**
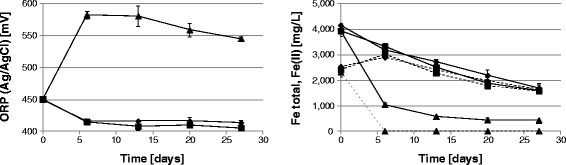
**Oxidation Reduction Potential (ORP) (Left panel) and total (continuous lines) and ferrous iron (dotted lines) (Right panel) in chalcopyrite bioleaching assays in the presence of chloride ion (3 g/L) at 50°C.** *Sulfobacillus thermosulfidooxidans* DSM 27601 strain Cutipay (▲); *Sulfobacillus acidophilus* DSM 10332 (♦); Control without inoculation (■).

In order to evaluate the relevance of strain Cutipay to enhance chalcopyrite dissolution under process conditions and compare it to the collection strain DSM 10332, chalcopyrite bioleaching tests were done with chloride ion. Copper recovery was periodically measured, showing 31% copper recovery in the abiotic control at the end of the test. The same effect was observed with *Sulfobacillus acidophilus*, being lower and reaching 26% recovery, probably inhibited by chloride ion. However, a strong increase in copper recovery was observed with strain Cutipay despite chloride present. As shown in Figure 2, chalcopyrite bioleaching was enhanced by strain Cutipay from the beginning, finishing at 44% on day 27. In the comparison with the collection strain *Sulfobacillus acidophilus* DSM 10332 and the abiotic control assays, strain Cutipay increased copper recovery in 18 and 13 percentage points which represented an increment of 69 and 42%, respectively, demonstrating its high potential for chalcopyrite bioleaching in the presence of chloride ion. Figure 3 shows that *Sulfobacillus thermosulfidooxidans* Cutipay was able to oxidize ferrous to ferric ions in the presence of chloride ion maintaining a high oxidation reduction potential, while *Sulfobacillus acidophilus* DSM10332 did not oxidize Fe(II) which is comparable to the control without inoculation. Previous results showed that both species have comparable iron oxidations rates, with same optimum pH of 1.7 and temperature (51°C) (Watling et al. [2008]), so the observed effect can be attributed only to chloride ion inhibition.

## Discussion

In this study a novel mixotrophic bacterium *Sulfobacillus thermosulfidooxidans* strain Cutipay (DSM 27601) was characterized for heavy metals and chloride resistance. Sodium and potassium chloride salts were separately used for chloride MIC testing, since the counter-ion (potassium or sodium) affects the inhibitory effect of anions (Suzuki et al. [1999]) and therefore the MIC. These results are only partially in agreement with our previous genomic analyses (Travisany et al. [2012]), supporting copper but not arsenic resistance. *Sulfobacillus thermosulfidooxidans* has been reported to be highly resistant to copper, nickel and zinc, while *Sulfobacillus acidophilus* to cobalt (Watling et al. [2008]). *Sulfobacillus thermosulfidooxidans* DSM9293 isolated from an ore deposit in Eastern Kazakhstan has been tested for sodium chloride resistance, starting to show inhibition at 7 g/L (4 g chloride/L) (Zammit et al. [2012]), while *Sulfobacillus* sp. TPY found in an hydrothermal vent in the Pacific Ocean grows in the presence of 20 g/L NaCl (12 g chloride/L) (Wang et al. [2012]). In this study *Sulfobacillus thermosulfidooxidans* Cutipay from an ore deposit in Chile was completely inhibited at 21 and 8 g/L potassium and sodium chloride, respectively, having chloride resistance comparable to previous reports for the same species, but below the marine strain TPY.

Based on the genome comparison, the chloride-resistant representative species have the Haloacid dehalogenase coding gene. Therefore, a possible mechanism of chloride resistance in sulfobacilli may be related to the biodegradation of toxic small chloro-organic acids formed in the presence of chloride, which are produced from the dissolution of gangue material in hydrometallurgical processes (Dopson et al. [2008]) that reacts with organic matter such as cell products or debris (Keppler et al. [2000]). To our knowledge no chloride resistance mechanism nor related gene has been identified up to date in acidophiles, and therefore this finding may provide new insights into extremophilic microorganisms behavior. On this matter, previous studies have shown the role of membrane and/or amino acid biosynthesis, efflux transporters and CO_2_ fixation as multifunctional responses to chloride exposure in acidophilic species (Zammit et al. [2012]). However these are general osmo-protection mechanisms observed in different microbial species (Zhou et al. [2013]; Paul [2013]).

Finally, the bioleaching tests showed that strain Cutipay resistance enhances chalcopyrite bioleaching at 50°C in the presence of chloride, based on its fast iron-oxidizing activity compared to the chloride sensitive strain *Sulfobacillus acidophilus* DSM 10332. Here, only in the case of strain Cutipay all the ferrous iron was completely oxidized. However, a major drop in total iron concentration was observed lowering from 4 to 1 g/L, due to the formation of iron precipitates such as jarosite, as visually observed only on strain Cutipay’s inoculated flasks (data not shown). No matter this precipitation and potential surface passivation, chalcopyrite bioleaching enhancement is clearly shown.

Summarizing, in this study a novel mixotrophic bacterium with resistance to chloride was presented, showing a genomic feature related to a potential new mechanism of chloride resistance and proven to enhance chalcopyrite bioleaching at 50°C in the presence of chloride, a relevant inhibitory element present in important industrial bioleaching processes.

## Additional file

## Electronic supplementary material

Additional file 1: Table S1.: Chalcopyrite Concentrate mineralogy. (DOCX 11 KB)

Below are the links to the authors’ original submitted files for images.

Authors’ original file for figure 1

Authors’ original file for figure 2

Authors’ original file for figure 3

Authors’ original file for figure 4
