# Supplementary material for: Sulfobacillus thermosulfidooxidans strain Cutipay enhances chalcopyrite bioleaching under moderate thermophilic conditions in the presence of chloride ion
Source: AMB Express. 2014 Dec 10;4:84. doi: 10.1186/s13568-014-0084-1 (PMC4884008; doi:10.1186/s13568-014-0084-1)
Supplement: Supplementary file 1 — Additional file 1: Table S1.: Chalcopyrite Concentrate mineralogy. (DOCX 11 KB) [file 13568_2014_84_MOESM1_ESM.docx]

Additional file 1

Table S1. Chalcopyrite Concentrate mineralogy.

| Minerals | % Weight |
| --- | --- |
| Chalcopyrite | 85,5 |
| Covellite | 0,11 |
| Tennantite | 0,11 |
| Pyrite | 3,84 |
| Marcasite | 0,08 |
| Pyrrhotite | 0,04 |
| Molybdenite | 0,02 |
| Sphalerite | 0,85 |
| Magnetite | 0,44 |
| Rutile | 0,13 |
| Gangue | 8,87 |
| Total | 99,99 |
