# Supplementary figures and images for: Sulfobacillus thermosulfidooxidans strain Cutipay enhances chalcopyrite bioleaching under moderate thermophilic conditions in the presence of chloride ion
Source: AMB Express. 2014 Dec 10;4:84. doi: 10.1186/s13568-014-0084-1 (PMC4884008; doi:10.1186/s13568-014-0084-1)

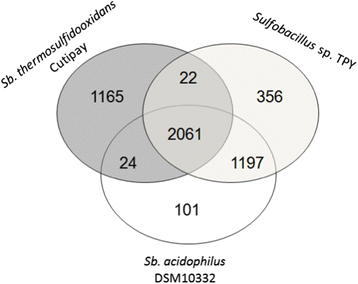

Supplement: Supplementary file 2 — Authors’ original file for figure 1 [file 13568_2014_84_MOESM2_ESM.gif]

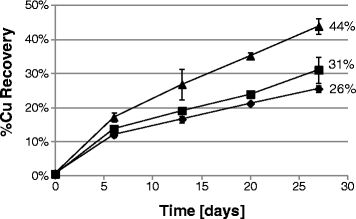

Supplement: Supplementary file 3 — Authors’ original file for figure 2 [file 13568_2014_84_MOESM3_ESM.gif]

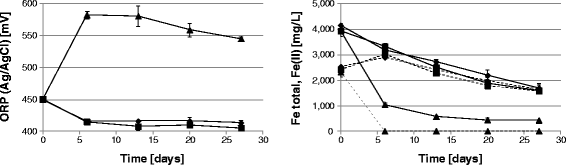

Supplement: Supplementary file 4 — Authors’ original file for figure 3 [file 13568_2014_84_MOESM4_ESM.gif]
