# Supplementary material for: Sulfobacillus thermosulfidooxidans strain Cutipay enhances chalcopyrite bioleaching under moderate thermophilic conditions in the presence of chloride ion
Source: AMB Express. 2014 Dec 10;4:84. doi: 10.1186/s13568-014-0084-1 (PMC4884008; doi:10.1186/s13568-014-0084-1)
Supplement: Supplementary file 5 — Authors’ original file for figure 4 [file 13568_2014_84_MOESM5_ESM.docx]

Table 1. Minimum Inhibitory Concentrations (MIC) for Inhibitory Elements Chloride, Copper and Arsenic.

| Moderate Thermophilic Species | Chloride (KCl) | Chloride (NaCl) | Cu(II) | As(III) |
| --- | --- | --- | --- | --- |
|  | [ppm] | | | |
| *Sulfobacillus thermosulfidooxidans* Cutipay (DSM 27601) | 10,000 | 5,000 | 3,000 | <100 |
| *Sulfobacillus acidophilus* (DSM 10332) | 3,000 | 1,000 | 1,000 | 1,000 |
